# Dust storms and cardiorespiratory emergency department visits in three Southwestern United States: application of a monitoring-based exposure metric

**DOI:** 10.1088/2752-5309/ad5751

**Published:** 2024-07-15

**Authors:** Claire Rowan, Rohan R D’Souza, Xiaping Zheng, James Crooks, Kirk Hohsfield, Daniel Tong, Howard H Chang, Stefanie Ebelt

**Affiliations:** 1 Department of Epidemiology, Rollins School of Public Health, Emory University, Atlanta, GA, United States of America; 2 Department of Biostatistics and Bioinformatics, Rollins School of Public Health, Emory University, Atlanta, GA, United States of America; 3 Division of Biostatistics and Bioinformatics, National Jewish Health, Denver, CO, United States of America; 4 Department of Atmospheric, Oceanic & Earth Sciences, George Mason University, Fairfax, VA, United States of America; 5 Gangarosa Department of Environmental Health, Rollins School of Public Health, Emory University, Atlanta, GA, United States of America

**Keywords:** climate, dust, PM, morbidity, emergency department visits

## Abstract

Climate change is projected to increase the risk of dust storms, particularly in subtropical dryland, including the southwestern US. Research on dust storm’s health impacts in the US is limited and hindered by challenges in dust storm identification. This study assesses the potential link between dust storms and cardiorespiratory emergency department (ED) visits in the southwestern US. We acquired data for 2005–2016 from eight IMPROVE (Interagency Monitoring of PROtected Visual Environments) sites in Arizona, California, and Utah. We applied a validated algorithm to identify dust storm days at each site. We acquired patient-level ED visit data from state agencies and ascertained visits for respiratory, cardiovascular, and cause-specific subgroups among patients residing in ZIP codes within 50 km of an IMPROVE site. Using a case-crossover design, we estimated short-term associations of ED visits and dust storms, controlling for temporally varying covariates. During 2005–2016, 40 dust storm days occurred at the eight IMPROVE sites. Mean PM_10_ and PM_2.5_ levels were three to six times greater on dust storm days compared to non-dust storm days. Over the study period, there were 2 524 259 respiratory and 2 805 925 cardiovascular ED visits. At lags of 1, 2, and 3 days after a dust storm, we observed 3.7% (95% CI: 1.0%, 7.6%), 4.9% (95% CI: 1.1%, 8.9%), and 5.0% (95% CI: 1.3%, 8.9%) elevated odds of respiratory ED visits compared to non-dust storm days. Estimated associations of dust storm days and cardiovascular disease ED visits were largely consistent with the null. Using a monitoring-based exposure metric, we observed associations among dust storms and respiratory ED visits. The results add to growing evidence of the health threat posed by dust storms. The dust storm metric was limited by lack of daily data; future research should consider information from satellite and numerical models to enhance dust storm characterization.

## 1. Introduction

Dust storms are an important contributor to environmental health concerns, with elevated dust levels causing a significant number of short and long-term health effects and affecting many components of health and stages of life globally [1, 2]. Dust storms occur when strong winds lift exposed soil particles (dust), typically under dry conditions, resulting in high concentrations of particulate matter (PM) and low visibility at affected locations. Dust emissions can take place anywhere in the world and their composition vary by area, but typically consist of crustal elements and their oxidants, such as silica, dialuminium dioxide, iron (III) oxide, and calcium oxide, as well as organic matter, including fungal and bacterial communities among others [3–5].

In the US, dust storms have shifted in scope and across regions over the past century. The well-known Dust Bowl in the 1930s was characterized by severe dust storms that impacted the Central Plains [6]. Currently in the US, dust storms are most common in the southwest, specifically the Great Basin, the Mohave, Sonoran, and Chihuahuan Deserts, the semiarid Columbia Plateau, Colorado Plateau, and the southern Great Plains [7]. Dust from international sources such as the Sahara Desert also impacts dust levels in the US due to transport via winds, causing higher dust concentrations beyond the origin area of the dust [8]. Climate change is expected to cause an increasingly dry climate in the subtropics, including in the southwestern US [9, 10]. As such, there are predicted increases in the concentration of dust and lengths of dust event seasons, lending great importance to understanding the health effects of the threat of dust storms [11, 12].

Global dust storm epidemiology has developed over the past decades, with many studies finding population-level impacts on total mortality, cardiovascular and respiratory mortality and hospital admissions, and pediatric asthma emergency department (ED) visits [13]. For example, in Taiwan, Asian dust storms have been linked to an increase in total and cardiovascular mortality [14]. Similar results have been observed from Saharan dust in Spain [15]. Khaniabadi *et al* [16] found an increase in respiratory mortality and hospital admissions for chronic obstructive pulmonary disease (COPD) in Iran from Middle Eastern dust storms [16]. Increases in dust storm-related respiratory, asthma, cardiovascular, and all-cause hospital admissions have been observed in Kuwait and Australia [17, 18]. Middleton *et al* [19] found higher rates of cardiovascular hospital admissions in Cyprus on dust storm days than non-dust storm days [19]. Asthma pediatric ED visits increased in Trinidad following African dust clouds [20].

Studies in the US have also found increases in total non-accidental and cardiovascular mortality, increases in total and respiratory intensive care unit admissions, and total hospitalizations on days of dust storms [21–23]. However, the literature on dust storm health impacts in the US is incomplete. A larger body of data is needed to better evaluate the magnitude of public health impacts and to prepare for current and future climate change. In particular, the role of dust storms in increasing the risks of ED visits and hospitalizations have not been fully characterized. Furthermore, enhanced dust storm identification metrics are needed for use in epidemiologic studies [24]. Previous studies have assessed exposure to dust storms using dust storm reports from the US National Weather Service (NWS) storm database [21, 22]. However inconsistencies in data collection across locations and time in this storm database creates a challenge for the use of these data in epidemiologic studies. Dust storms identified from ground monitors that provide an accurate measure of local air quality conditions are crucial for developing accurate exposure-response functions.

To address these gaps, we conducted a multi-year study for the period 2005–2016 to assess the links between dust storms and cardiorespiratory ED visits in three southwestern states of the US (Arizona (AZ), California (CA), and Utah (UT)) that are frequently impacted by dust storms. We applied a novel and validated method for identifying dust storm days using routine ground air pollution monitoring data [25].

## 2. Methods

### 2.1. Dust storm and other air quality data

**Figure 1.**
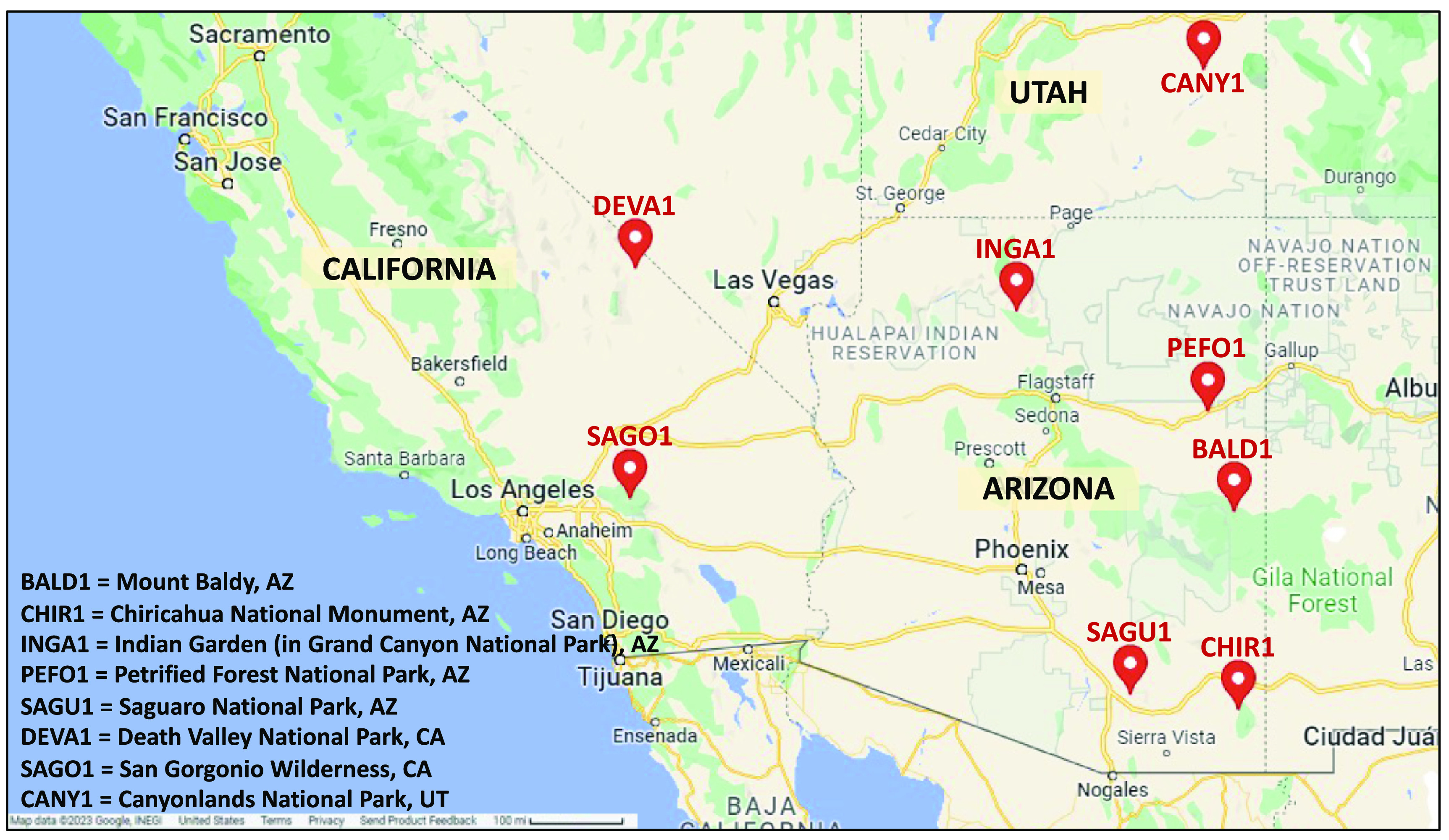
Map of IMPROVE monitoring sites [26].

The Interagency Monitoring of PROtected Visual Environments (IMPROVE) program, operated by US federal agencies, provides air pollution monitoring in US National Parks, Monuments, and Wilderness Areas. IMPROVE sites collect detailed 24-h average data on particle mass and composition every 3 or 6 d depending on the site. For this study, we acquired data from eight IMPROVE sites in California (2 sites), Arizona (5 sites), and Utah (1 site) for the period 1 January 2005 to 31 December 2016 (figure 1).

Using an algorithm developed by Tong *et al* [25], also applied by Hohsfield *et al* [24], we compiled a database of dust storm days at each IMPROVE site [24, 25]. Leveraging the detailed particle composition data measured by the IMPROVE monitors, the algorithm applies five criteria that allows for the differentiation between dust and other air quality events such as wildfire smoke, as well as differentiation of natural and anthropogenic origins of dust. The criteria include: (1) high PM_10_ and PM_2.5_ concentrations, (2) a low ratio of PM_2.5_ to PM_10_, (3) high concentrations of crustal elements [including silicon (Si), calcium (Ca), potassium (K), iron (Fe), and titanium (Ti)], (4) low concentrations of anthropogenic pollutants [including arsenic (As), zinc (Zn), copper (Cu), lead (Pb), sulfate (SO_4_^2−^), nitrate (NO_3_^−^), organic carbon (OC), and elemental carbon (EC)], and (5) low enrichment ratios of specific anthropogenic pollutants (Zn, Cu, Pb, and K as indicators of industrial operations and biomass burning) relative to Earth’s crust [25]. This algorithm can be considered a ‘gold standard’ of dust storm classification in the US as the dust storms are measured, and the system is trained and validated against satellite imagery of confirmed dust events. The resulting dust storm exposure dataset included a dichotomous indicator variable identifying days without (0) and days with a dust storm (1) at each IMPROVE site; days with no IMPROVE measurements were coded as missing for the dust storm indicator.

Other air quality data of interest for use as covariates in epidemiologic models included daily ZIP code-level meteorological variables (maximum temperature and mean dew point temperature) and criteria pollutants [24-hr average fine PM (PM_2.5_), 1-h maximum nitrogen dioxide (NO_2_), 8 h maximum ozone (O_3_)]. Meteorological data for 2005–2016 were acquired from Daymet [27]. Data on air pollutants for 2005–2016 were obtained from ensemble prediction of several machine learning algorithms available through the NASA Socioeconomic Data and Applications Center [28, 29].

### 2.2. Emergency department visit data

Data on ED visits were collected from patient-level hospital billing records acquired for Arizona (Arizona Hospital Discharge Data Set, Bureau of Public Health Statistics, Arizona Department of Health Services; data available: 1 July 2010–31 December 2016), California (California Health and Human Services Agency, Office of Statewide Planning and Development; data available 1 January 2005–31 December 2016), and Utah (Utah Department of Health & Human Services: Utah Health Data Committee; data available: 1 January 2005–31 December 2016). ED visits included both outpatient encounters (i.e. seen in the ED and discharged) and inpatient encounters (i.e. admitted to the hospital from the ED). Variables used in this analysis included the admission date, primary and secondary International Classification of Diseases (ICD) diagnosis codes, and ZIP code of the patient’s residence.

ICD diagnosis codes (version 9 prior to 1 October 2015; version 10 after and including 1 October 2015) in any diagnosis field (i.e. primary or secondary) were used to identify cardiorespiratory outcomes of interest: all respiratory diseases (RD; ICD-9 codes 460-519; ICD-10 codes J00–J99), asthma (ICD-9 code 493; ICD-10 code J45), COPD (ICD-9 codes 491, 492, 496; ICD-10 codes J41–J44), and all cardiovascular diseases (CVD; ICD-9 codes 390–459, ICD-10 codes I00–I99), ischemic heart disease (IHD; ICD-9 codes 410-414, ICD-10 codes I20–I25), cardiac dysrhythmia (DYS; ICD-9 codes 427, ICD-10 codes I47–I49), and congestive heart failure (CHF; ICD-9 codes 428, ICD-10 codes I42, I50, I51).

For each outcome, ED visits were included in the analysis if the patient residential ZIP code was wholly or partially within 50 km of an IMPROVE site. Given the size of ZIP codes in these states, the median distance from each site to the farthest boundary of each site’s study area was 145 km (supplemental figure S1). Dust storms identified by the Tong *et al* [25] method are rather large and long lasting, with 24-h average PM_10_ concentrations usually over 40 *μ*g m^−3^. In addition, wind speeds during local dust storms are usually high, allowing dust plumes to cover a large geographical scope; for example, analysis of satellite imagery has identified dust storms covering areas over 50 000 km^2^ [30], thus justifying the 50 km proximity buffer. Two patient ZIP codes fell within 50 km of two different IMPROVE monitors; in these cases, the ZIP code was paired with its closest monitor. A proximity buffer of 15 km was also considered in secondary analyses, to capture a more accurate exposure assessment but with reduced power due to lower ED visit numbers within a smaller buffer area.

### 2.3. Application of case-crossover analysis

The goal of this study was to estimate associations between dust storm days and ED visits. Given the unique ability for a case-crossover design to study acute health events [31, 32], it is well suited for the application of dust storms, which are relatively short events with the potential to cause health events within hours to days.

Typically, case-crossover epidemiologic studies assessing short-term effects of air pollution use monthly study frames, in which case days are identified as the days on which an outcome occurred, and control days are selected for each case day by matching on year, month, and day of week (i.e. if a case day was a Tuesday in August 2015, the control days would be all other Tuesdays in August 2015). This control day selection enables control of long-term and seasonal trends as well as day of week by design. In the current study, since IMPROVE monitoring data were available only on a 1-in-3 or 1-in-6 d basis, matching on day of week was not possible as exposure data were not available on the same day of week for every week of each month. As such, we developed customized strata for the 1 January 2005–31 December 2016 period in which each stratum consisted of 4 d at 6 d intervals from each other to align with exposure data availability. ED visits (cases) were assigned a stratum based on their date of visit, and controls days were selected for each case as the other 3 d in the corresponding stratum. Thus, control days were all within a 24 d window, allowing control of long-term and seasonal trends by design. In addition, the timing of controls days was on average both before and after the case day, which is the desired property of a time-stratified design [33].

The dust storm indicator data were then merged into the case-crossover dataset by IMPROVE site and date. Each case and their matching control days were assigned dust storm indicator values from the nearest IMPROVE site on the same day as the case or control (lag 0) and on the 5 d leading up to the case or control (lags 1–5). Other air quality data were additionally linked to ED visits by ZIP code and date.

To estimate associations of dust storm days and ED visits, we conducted conditional logistic regression analyses matched by case-crossover strata. The models included the dust storm indicator on the day of each case and control (lag 0); in separate models, the effects of dust storms of up to 5 d prior to the case and control were assessed. All models were single-day lag models. Models considering multiple lag days simultaneously (e.g. distributed lag models) could not be assessed given the every-3-d or every-6-d nature of the exposure data. All strata with missing dust storm information were dropped from analyses (i.e. only those strata aligning with the 1-in-3 or 1-in-6 d IMPROVE monitoring schedule ultimately provided information to the analyses). The models additionally included indicator variables for day-of-week and federal holidays. Meteorology was controlled using linear, squared, and cubic terms for daily maximum temperature and mean dew point temperature for the same lag as the dust storm indicator being evaluated. In primary analyses, we ran this main model for each dust storm lag (lag days 0–5) and each main outcome (RD, CVD) for all states combined and stratified by state (AZ, CA, UT). In secondary analyses, we considered all state models stratified by cardiorespiratory subgroups (asthma, COPD, CVD, IHD, DYS, CHF). In sensitivity analyses, we restricted cases to those living in ZIP codes within 15 km of an IMPROVE site, and we adjusted for daily PM_2.5_, NO_2_, and O_3_ using the same lag as the dust storm indicator being evaluated.

All analyses were conducted in SAS V9.4 (SAS Institute, Inc., Cary, NC, USA) and R Statistical Software (v4.1.3; R Core Team 2022).

## 3. Results

### 3.1. IMPROVE monitoring data on dust storm vs. non-dust storm days

The study period (i.e. 7/2010–2016 in Arizona, and 2005–2016 in California and Utah) consisted of 25 029 site-days across the eight IMPROVE monitoring sites. With the non-daily IMPROVE sampling schedule, 7048 site-days had a dust storm indicator and of which 40 site-days were identified as dust storm days. As a check of the algorithm used to identify dust storm days [25], we assessed the concentrations of different PM size classes on dust storm vs. non-dust storm days. As expected, we observed increased PM concentrations on days indicated as dust storm days compared to those indicated as non-dust storm days (table 1). For example, PM_10_ and PM_2.5_ concentrations on days with dust storms were on average 50.8 *µ*g m^−3^ (6.0 times) and 9.1 *µ*g m^−3^ (2.6 times) higher, respectively, than those on non-dust storm days.

**Table 1.** PM mass and selected PM component concentrations (*µ*g m^−3^) on dust vs. non-dust storm days for eight IMPROVE monitoring sites in Arizona (7/2010–2016), California (2005–2016), and Utah (2005–2016).

Particulate matter	Dust storm days			Non-dust storm days			Difference^c^	Relative difference^d^
	N^a^	Mean	SD	N^b^	Mean	SD		
PM_10_	40	59.4	30.9	6578	8.5	6.8	50.8	6.0
PM_10–2.5_	40	46.8	25.4	6160	5.0	4.7	41.8	8.4
PM_2.5_	40	12.5	6.8	6594	3.5	2.7	9.1	2.6
PM_2.5_ components:								
Silicon (Si)	40	2.0	1.1	6598	0.19	0.20	1.8	9.6
Nitrate (NO_3_^−^)	40	0.32	0.26	6623	0.36	0.69	−0.03	−0.09
Sulfate (SO_4_^2−^)	40	0.85	0.53	6623	0.62	0.45	0.23	0.37
Elemental carbon (EC)	39	0.036	0.031	6233	0.12	0.17	−0.08	−0.70
Organic carbon (OC)	39	0.65	0.37	6233	0.61	1.10	0.04	0.07

^a^ Dust storm days = number of site-days with IMPROVE monitoring and dust storm indicated.

^b^ Non-dust storm days = number of site-days with IMPROVE monitoring and no dust storm indicated.

^c^ Difference = Mean_DustStormDays_−Mean_NonDustStormDays._

^d^ Relative difference = (Mean_DustStormDays_−Mean_NonDustStormDays_)/Mean_NonDustStormDays._

We also assessed the concentrations of representative PM_2.5_ components used by the Tong *et al* [25] algorithm to demonstrate that the algorithm worked as it should to identify dust storm days in our dataset (table 1) [25]. As expected, PM_2.5_ silicon, a crustal element and key natural component of dust, was 2.0 *µ*g m^−3^ (9.6 times) higher on dust compared to non-dust storm days. And as anticipated, we did not observe a difference in the concentrations of major anthropogenic PM_2.5_ components (nitrate, sulfate, elemental and organic carbon) between dust storm and non-dust storm days, with relative differences less than 1.

### 3.2. Summary statistics of analytic database

Table 2 presents the numbers of dust storm days by IMPROVE site, and corresponding summary statistics of other air quality data and numbers of ED visits for each outcome. Over the study period, 2 524 259 ED visits for respiratory diseases and 2 805 925 ED visits for cardiovascular diseases were observed among patients living in ZIP codes within 50 km of the eight monitoring sites. Among the five sites in Arizona, two sites (BALD1 and PEFO1) experienced no dust storms. The Arizona SAGU1 site experienced the greatest number of dust storm days (*n* = 10); this site is located in Saguaro National Park near Tucson, AZ (figure 1) and also had highest numbers of ED visits (500 371 RD ED visits, 667 869 CVD ED visits) among Arizona sites. In California, while the DEVA1 site experienced the greatest number of dust storm days (*n* = 12), this site is located Death Valley National Park (figure 1) and had few ED visits (98 RD and 112 CVD ED visits). The SAGO1 site in California, with only four dust storm days observed, is located in the San Gorgonio Wilderness near San Bernardino and Riverside (figure 1) and contributed the most ED visits to the analysis: 1 921 334 RD ED visits (76% of total) and 2 033 779 CVD ED visits (72% of total).

**Table 2.** Summary statistics of dust storm days, other air quality parameters, and cardiorespiratory ED visits at each IMPROVE monitoring site.

IMPROVE sites	# of Dust storm days^a^	Other air quality parameters (daily mean (SD))^b^					# of Respiratory disease ED visits^c^			# of Cardiovascular ED visits^d^			
		Max temp (°C)	Mean dewpoint temp (°C)	24-h Avg PM_2.5_ (*µ*g m^−3^)	1-h Max NO_2_ (ppb)	8-h Max O_3_ (ppb)	RD	ASTHMA	COPD	CVD	IHD	DYS	CHF
ARIZONA (1 July 2010–31 December 2016)													
BALD1	0	19.5 (8.2)	−1.6 (7.6)	2.7 (1.8)	8.6 (9.7)	47.2 (7.8)	30 356	7477	6640	36 872	8605	5526	4303
CHIR1	7	25.4 (7.6)	2.0 (8.6)	2.7 (1.7)	14.3 (5.9)	49.7 (8.4)	27 842	4023	5418	32 593	7573	5832	4255
INGA1	0	20.3 (9.5)	−0.2 (6.6)	1.6 (1.6)	0.3 (0.6)	51.2 (7.1)	6909	2150	908	8563	1647	1206	739
PEFO1	1	21.5 (9.3)	−3.3 (7.7)	3.0 (1.6)	11.4 (4.6)	48.8 (8.4)	28 036	4625	2489	16 704	3343	2329	2064
SAGU1	10	28.6 (7.8)	5.5 (7.8)	3.7 (1.8)	20.3 (8.0)	49.2 (8.8)	500 371	167 971	91 974	667 869	149 665	130 158	74 933
CALIFORNIA (1 January 2005–31 December 2016)													
DEVA1	12	26.3 (9.8)	0.0 (6.5)	3.6 (2.8)	12.3 (6.6)	49.6 (10.7)	98	17	24	112	25	18	8
SAGO1	4	24.9 (7.8)	1.4 (4.0)	4.2 (3.4)	18.2 (9.6)	54.3 (14.8)	1 921 334	543 966	304 156	2 033 779	406 367	327 560	326 965
UTAH (1 January 2005–31 December 2016)													
CANY1	6	18.2 (10.8)	−2.5 (6.7)	2.7 (1.8)	10.3 (5.6)	50.4 (7.8)	9313	1895	1642	9433	1614	1782	1493
Total	**40**						**2 524 259**	**732 124**	**413 251**	**2 805 925**	**578 839**	**474 411**	**414 760**

^a^ Dust storm days identified at IMPROVE monitoring sites.

^b^ Obtained for ZIP codes located wholly or partially within 50 km of the IMPROVE monitoring sites; calculated as the daily mean of parameter across all ZIP codes.

^c^ RD = all respiratory disease, COPD = chronic obstructive pulmonary disease.

^d^ CVD = all cardiovascular disease, IHD = ischemic heart disease, DYS = dysrhythmia, CHF = congestive heart failure.

### 3.3. Associations of dust storms and respiratory ED visits

**Figure 2.**
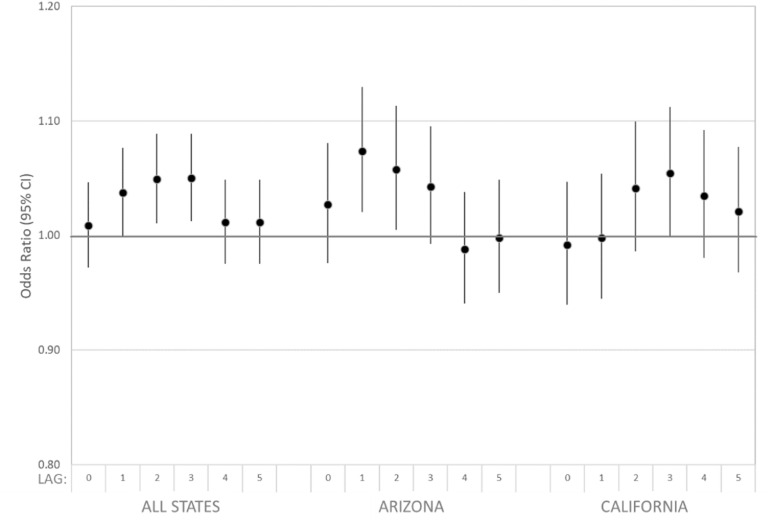
Estimated associations of all respiratory disease ED visits and dust storm days identified at eight IMPROVE sites overall and by state in Arizona (7/2010–2016), California (2005–2016), and Utah (2005–2016) among patients residing in ZIP codes within 50 km of any site. Case-crossover model covariates included: indicator variables for day-of-week and federal holidays; and linear, squared, and cubic terms for daily maximum temperature and mean dew point temperature for the same lag as the dust storm indicator being evaluated.

Estimated associations of dust storm days and all respiratory disease ED visits are presented in figure 2 and supplemental table S1. Overall, dust storms were associated with respiratory ED visits occurring 1–3 d later. For example, we observed odds ratios of 1.037 (95% CI: 1.000, 1.076), 1.049 (95% CI: 1.011, 1.089), and 1.050 (95% CI: 1.013, 1.089) for respiratory ED visits on 1, 2, and 3 d after a dust storm compared to non-dust storm days, respectively. Patterns of associations across lag days were similar when analyses were stratified by state, with elevated odds ratios observed in Arizona and California at lag days 1–3. Note that the low number of dust storm days prevented state-specific analyses for Utah.

**Figure 3.**
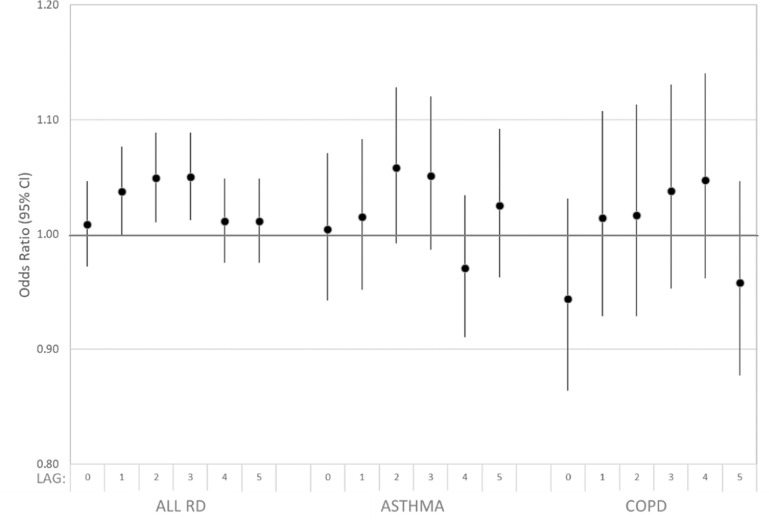
Estimated associations of all respiratory disease, asthma, and COPD ED visits and dust storm days identified at eight IMPROVE sites in Arizona (7/2010–2016), California (2005–2016), and Utah (2005–2016) among patients residing in ZIP codes within 50 km of any site. Case-crossover model covariates included: indicator variables for day-of-week and federal holidays; and linear, squared, and cubic terms for daily maximum temperature and mean dew point temperature for the same lag as the dust storm indicator being evaluated.

In secondary analyses, we considered overall models stratified by the respiratory subgroups, asthma and COPD (figure 3, supplemental table S1). The pattern and magnitudes of observed odds ratios [e.g. at lag day 2, the OR for asthma was 1.058 (95% CI: 0.993, 1.128)] were similar to those for all respiratory disease, albeit with considerably wider confidence intervals given the low case numbers.

In sensitivity analyses, we restricted cases to those living in ZIP codes within 15 km of an IMPROVE monitoring site for all respiratory ED visits. The total number of respiratory ED visits among patients within the 15 km buffer (338 762 ED visits) was 13% of those within the 50 km buffer (supplemental table S2). Estimated associations among cases restricted to the closer proximity buffer were consistent with the null, with effect estimates of similar or weaker magnitude than those obtained with cases pulled from the primary 50 km buffer (supplemental figure S2). In addition, to assess potential confounding by criteria pollutants, we compared primary and secondary main model results with those adjusted for PM_2.5_, NO_2_, and O_3_ using the same lag as the dust storm indicator being evaluated. As shown in supplemental table S1, adjustment by these pollutants did not meaningfully change dust storm odds ratios or observed patterns of effect across lag days.

### 3.4. Associations of dust storms and cardiovascular ED visits

**Figure 4.**
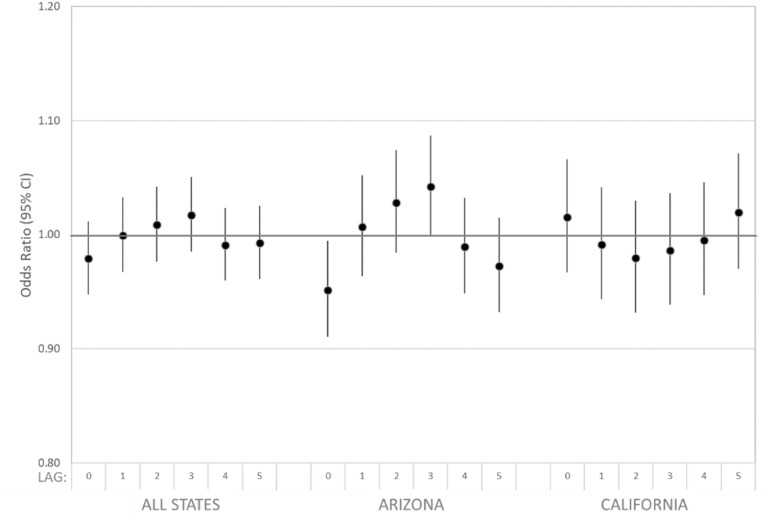
Estimated associations of all cardiovascular disease ED visits and dust storm days identified at eight IMPROVE sites overall and by state in Arizona (7/2010–2016), California (2005–2016), and Utah (2005–2016) among patients residing in ZIP codes within 50 km of any site. Case-crossover model covariates included: indicator variables for day-of-week and federal holidays; and linear, squared, and cubic terms for daily maximum temperature and mean dew point temperature for the same lag as the dust storm indicator being evaluated.

**Figure 5.**
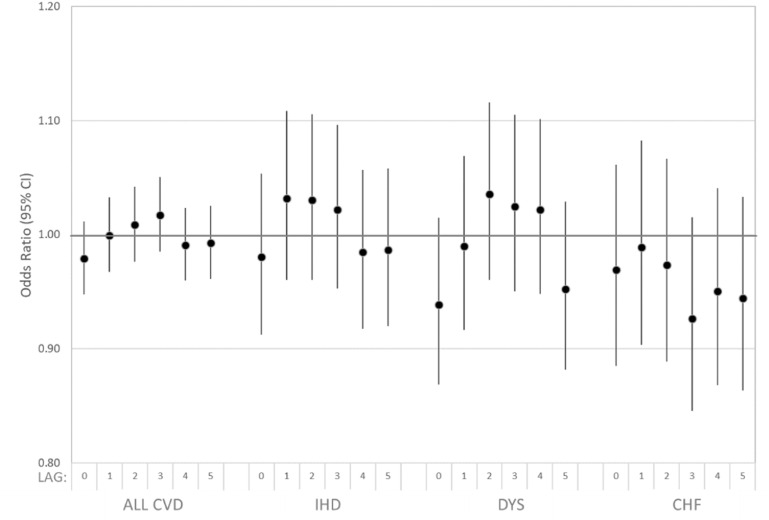
Estimated associations of all cardiovascular disease (CVD), ischemic heart disease (IHD), dysrhythmia (DYS), and congestive heart failure (CHF) ED visits and dust storm days identified at eight IMPROVE sites in Arizona (7/2010–2016), California (2005–2016), and Utah (2005–2016) among patients residing in ZIP codes within 50 km of any site. Case-crossover model covariates included: indicator variables for day-of-week and federal holidays; and linear, squared, and cubic terms for daily maximum temperature and mean dew point temperature for the same lag as the dust storm indicator being evaluated.

Estimated associations of dust storm days and all cardiovascular disease ED visits were largely consistent with the null (figure 4, supplemental table S3). The pattern of effect across lag days suggested elevated odds at 3 d after a dust storm, driven by Arizona data. For example, at a lag of 3 d after a dust storm, we observed an odds ratio of 1.042 (95% CI: 0.999, 1.087) for all cardiovascular ED visits compared to non-dust storm days in Arizona. This result was sensitive to adjustment by co-pollutants, after which we observed an odds ratio of 1.026 (95% CI: 0.980, 1.074) (supplemental table S3). Estimated associations for cardiovascular subgroups (IHD, DYS, CHF) were similarly largely consistent with the null (figure 5), as were associations among cases restricted to those living in ZIP codes within 15 km of an IMPROVE monitoring site (supplemental table S2 and figure S3).

## 4. Discussion

We conducted a multi-year study during 2005–2016 to assess the links between dust storms and cardiorespiratory ED visits in three southwestern US states (Arizona, California, and Utah). We applied a novel and validated method for identifying dust storm days using routine ground monitoring air pollution data from eight IMPROVE network sites [25]. Dust storm days were more frequently identified in Arizona (*n* = 18) and California (*n* = 16) during the 2005–2016 period than in Utah (*n* = 6). As expected, dust storm days had considerably higher concentrations of PM_10_, PM_2.5_ and certain PM_2.5_ elements (e.g. Si) than non-dust storm days. Overall, we observed consistent associations of dust storms in the previous 1–3 d and respiratory ED visits that were not confounded by NO_2_, O_3_, or PM_2.5_. Associations were generally consistent with the null for cardiovascular ED visits and cardiovascular subgroups.

This study adds to the growing global dust storm epidemiological literature [13], and fills a gap in US-based studies of dust storms and health [21–23]. Our results suggest that the complex mixture of PM from dust storms has implications for respiratory health. Among previous US-based studies, Crooks *et al* [21] assessed total non-accidental and cardiovascular mortality [21], Rublee *et al* [22] examined ICU admissions [22], and Peng *et al* [23] considered respiratory hospitalizations [23]. To our knowledge, this is the first study to focus specifically on respiratory and cardiovascular ED visits. Despite the different health endpoints assessed, the effect estimates observed in this analysis (i.e. ∼5% increased odds of respiratory ED visits 2–3 d after dust storms) are of a similar magnitude and follow a similar lag structure as those observed by the other US-based studies of dust storms. For example, Crooks *et al* [21] observed 6%–7% increases in total non-accidental mortality on the 2–3 d following dust storms. Rublee *et al* [22] found a 4.8% increase in total ICU admissions and a 9.2% increase in respiratory ICU admissions the day of a dust storm. Peng *et al* [23] found a 10% increase in respiratory hospitalizations on days with versus without dust storms. Together, these results add to our understanding that dust storms, which acutely reduce visibility and can cause nuisance and economic hazards, also have important public health impacts through a variety of health care utilization metrics that add to the burden experienced by the population.

There are several strengths to highlight for this analysis, including the multi-year study period in three US states where dust storms are of concern, the assessment of ED visits that have been an understudied health endpoint with dust storms to date, the use of a validated dust storm metric based on continuous ground-level monitoring data, and inclusion of criteria pollutants (PM_2.5_, NO_2_, and O_3_) that themselves have been linked with adverse cardiorespiratory health [34–36]. It is important to note that the total number of dust storm days identified in this study (*n* = 40) was much lower than the number of dust storms observed in other US-based studies with slightly larger observation periods. For example, Crooks *et al* [21] found 209 dust storms during 1993–2005 and Rublee *et al* [22] observed 967 dust storms during 2000–2015 using dust storm events captured by the NWS storm database, which includes reports from the insurance industry, damage surveys, and more [21, 22]. Peng [23] found 142 dust storms occurring between 2000 and 2005 using an NWS analysis of hourly weather observers’ records in El Paso, Texas [23]. The dust storm metrics used in these prior studies have limitations in how dust events were reported, defined, and identified and it was a motivation for using the IMPROVE monitoring-based metric applied in current study.

We assumed that dust storm days indicated by the IMPROVE monitoring data were representative of ZIP codes within 50 km of a given site. Measurement error in the exposure usually causes a bias to the null, and as such we interpret the observed associations of dust storm days and respiratory ED visits as conservative estimates of effect. In sensitivity analyses, we restricted to a closer proximity buffer of 15 km, considering that the dust storm days indicator could be more representative of exposures among patients living closer to the monitoring sites. While the 15 km buffer captured fewer case counts and thus had more limited statistical power compared to the 50 km buffer, the estimated magnitudes of effect for the 15 km buffer were similar or weaker to those from the 50 km buffer. Altogether, the comparison suggests that our use of a 50 km buffer provided a reasonable balance of exposure representativeness and statistical power.

There are several limitations to acknowledge. The IMPROVE monitoring network, while providing highly detailed PM_2.5_ speciation, does not provide speciation for larger PM size fractions, such as PM_10_ or total suspended particulates. There is a possibility that our dust storm days were under-counted given that our algorithm did not include information on these larger size fractions that can also contain dust particles [30]. In addition, the IMPROVE monitoring network typically only provides data on a 1-in-3 or 1-in-6 d sampling schedule. Our dust storm indicator was also limited to the 1-in-3 or 1-in-6 d schedule and thus had much missingness over the study period that prevented assessment of longer lag structures as well as moving average (cumulative) effects over several days. Ultimately, the individual lag day assessment that was conducted, each contained a unique case day set; this caused some instability in patterns of effect across lag days, in particular for secondary analyses with lower overall case counts (e.g. by state, by cardiorespiratory subgroup). In addition, the majority of IMPROVE monitors are purposefully sited in rural areas, including national or state parks. Thus, observed associations in this study do not capture the impact of dust storms on large population centers in Arizona, California, or Utah. And, given that rural areas contain fewer people, with fewer ED visits, our secondary and sensitivity analyses lacked power to detect associations. Conversely, the locations of IMPROVE sites in rural areas do by design prevent confounding by urban air pollutants. Future work will incorporate satellite and models for enhanced dust storm characterization.

## 5. Conclusion

The widely observed increasing frequency of dust storms globally has been linked to climate change through the desertification of soil [9]. Epidemiological studies point to significant public health impacts due to dust storms [1]. In this multi-year study in the southwestern US applying a monitoring-based exposure metric, we observed strong associations of dust storms in the previous 1–3 d and respiratory ED visits. With the observed increase in global temperatures, and increasing frequency and intensity of dust storms, climate and health mitigation and prevention measures should include consideration of dust storm health impacts.

## Data Availability

As part of the data use agreement with individual states, we are prohibited from sharing the ED visits data. However, similar data used in this analysis can be directly requested from the corresponding health departments or agencies. The Daymet meteorological data are publicly available at https://daymet.ornl.gov/. The IMPROVE data are publicly available at http://vista.cira.colostate.edu/Improve/improve-data/. The data that support the findings of this study are available upon reasonable request from the authors.
